# Supplementary material for: 3D Spheroid Configurations Are Possible Indictors for Evaluating the Pathophysiology of Melanoma Cell Lines
Source: Cells. 2023 Feb 27;12(5):759. doi: 10.3390/cells12050759 (PMC10000690; doi:10.3390/cells12050759)
Supplement: Supplementary file 1 [file cells-12-00759-s001.zip › cells-2126729-supplementary.pdf]

Table S1. Primers for qPCR.

		Sequence	Exon Location	RefSeq Number
KRAS	probe	5'-/56-FAM/AGGTGGTGG/ZEN/CTGATGCTTTGAACA/3IABkFQ/-3'	6-6	NM_033360
	Primer2	5'-CCTACTGTCGCTAATGGATTGG-3'		
	Primer1	5'-TGCCCTACATCTTATTTCCTCAG-3'		
SOX2	probe	5'-/56-FAM/CACCTACAG/ZEN/CATGTCCTACTCGCA/3IABkFQ/-3'	1-1	NM_003106
	Primer2	5'-CTTGACCACGAACCCAT-3'		
	Primer1	5'-GTACAACTCCATGACCAGCTC-3'		
STAT3	probe	5'-/56-FAM/AGCTGCACC/ZEN/TGATCACCTTTGAGAC/3IABkFQ/-3'	14-16	NM_213662
	Primer2	5'-AGGCATTTGGCATCTGACAG-3'		
	Primer1	5'-TGCTTCCCTGATTGTGACTG-3'		
BRAF	probe	5'-/56-FAM/AGGGAAAGT/ZEN/GGCATGGTGATGTGG/3IABkFQ/-3'	11-12	NM_004333
	Primer2	5'-CTGAGGTGTAGGTGCTGTC-3'		
	Primer1	5'-TGATGGGCAGATTACAGTGG-3'		

Table S2. List of the top 10 DEGs that are up-regulated or down-regulated between WM266-4 and SK-mel-24.

Expr Log Ratio ↑		Expr Log Ratio ↓	
molecules	value	molecules	value
HLA-DQA1	16.233	BIRC7	-13.356
HLA-DQB1	15.287	PNMA8A	-12.708
XXbac-BPG248L24,12	15.224	ITGAX	-12.644
IL1A	15.165	TRIL	-12.609
HLA-DRB1	12.044	GYG2	-12.535
RPS4Y1	14.401	VWA1	-12.311
GFRA1	13.533	TNFRSF10D	-12.257
UCHL1	13.429	TPSAB1/TPSB2	-12.167
DDX3Y	13.409	TUBB4A	-12.118
KRT81	12.776	CYBA	-12.166

Table S3. List of the top disease and disorders and molecular and cellular functions.

name	p-value range
cancer	2.60E-20 - 4.67E-288
organismal Injury and Abnormalities	2.60E-20 - 4.67E-288
endocrine system disorders	3.08E-22 - 4.65E-191
gastrointestinal diseases	1.32E-20 - 1.31E-190
neurological diseases	5.60E-21 -5.38E-132

Figure S1. GO enrichment analysis and Ingenuity Pathway Analysis (IPA) of DEGs of 2D WM266-4 and SK-mel-24.

Up-regulated and down-regulated mRNA-enriched biological functions were validated by GO analysis.

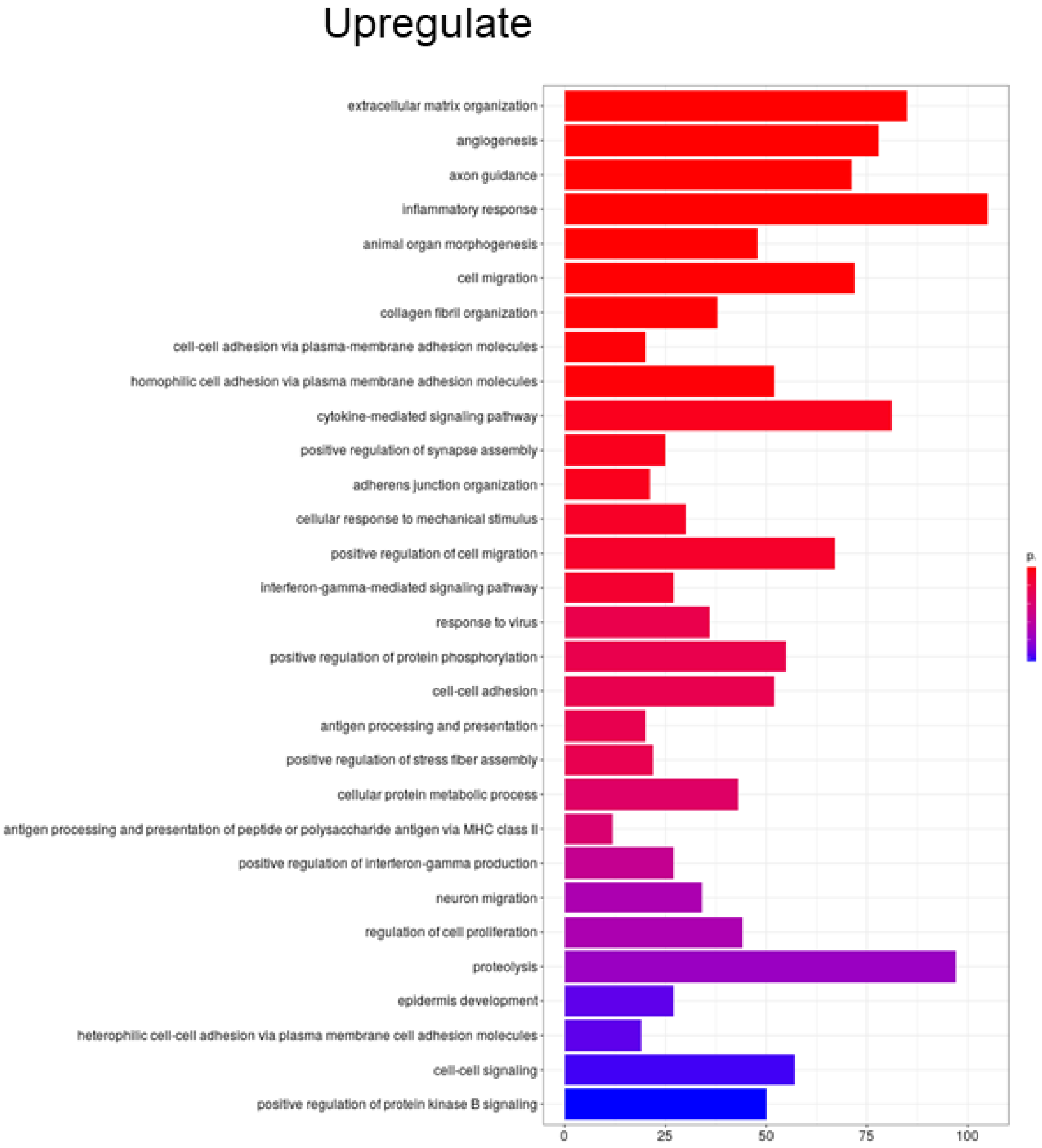

Downregulate

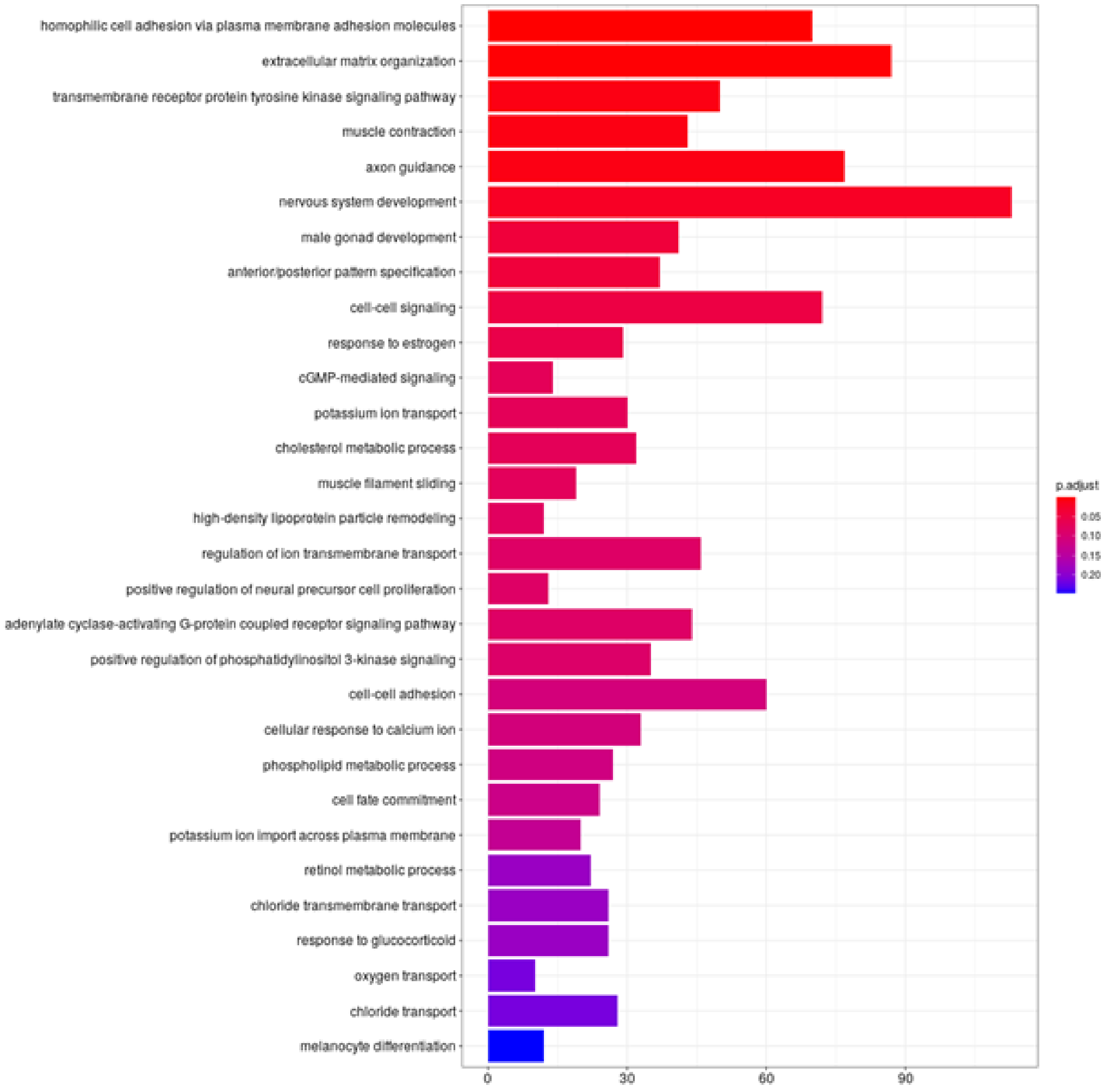

Figure S2. Causal networks related to KRAS and SOX2. Prediction legend is shown in Fig. S3.

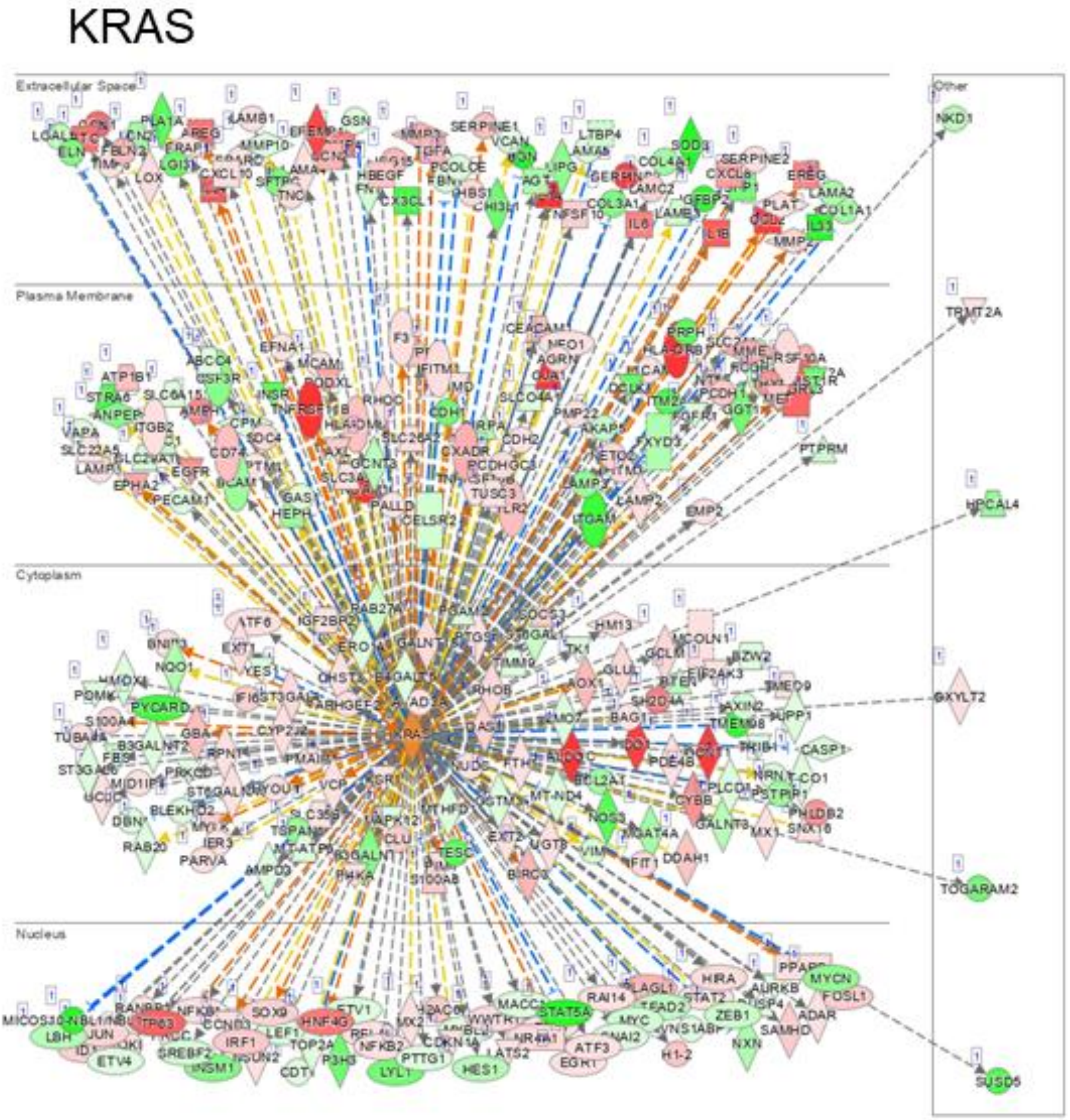

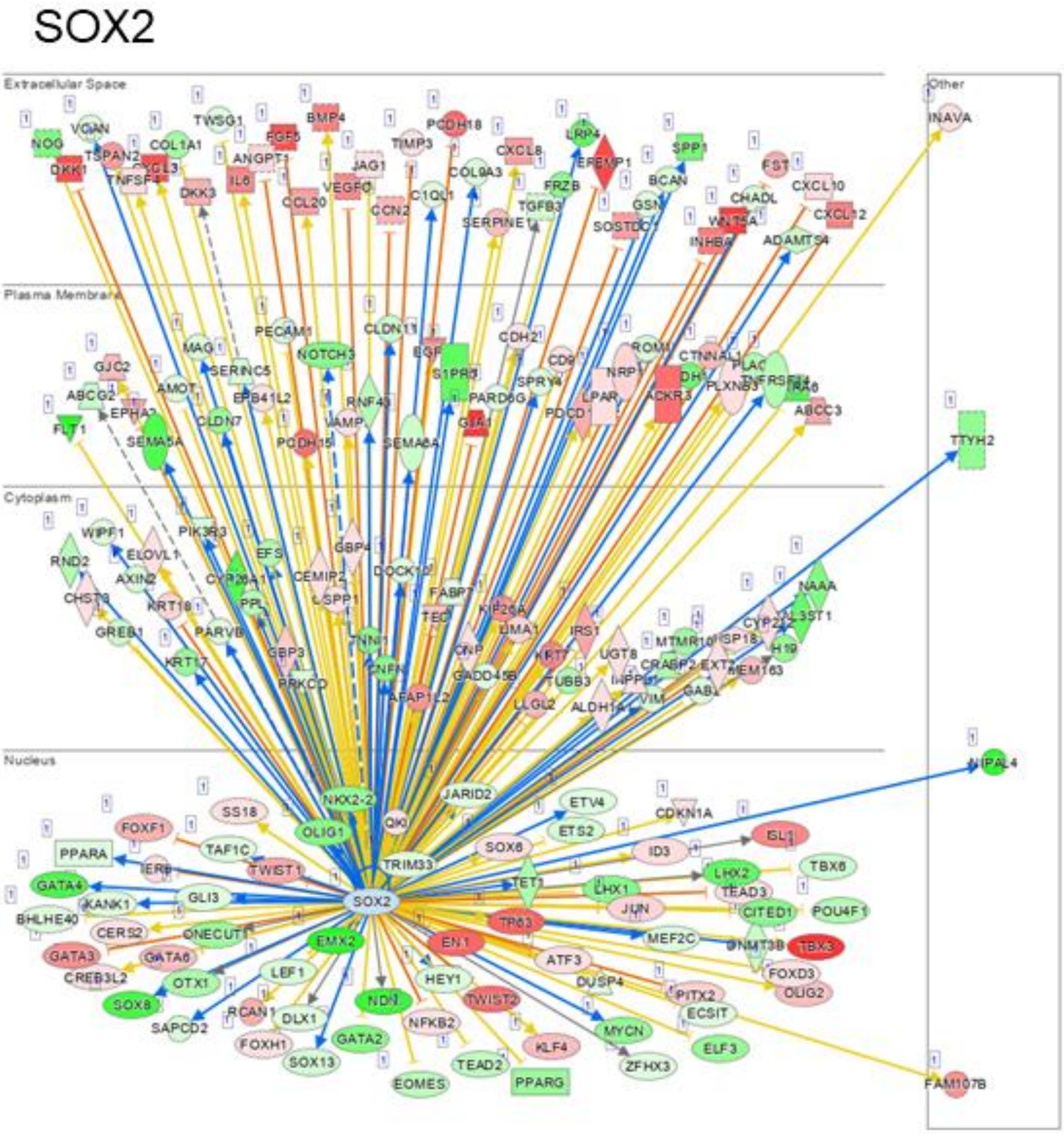

Figure S3. Prediction Legend.

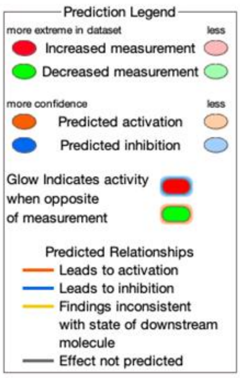

Figure S4. The comparison of the effects of two different siRNAs for KRAS or SOX2 on the mitochondrial and glycolytic functions of 2D cultured SK-mel-24 cells.

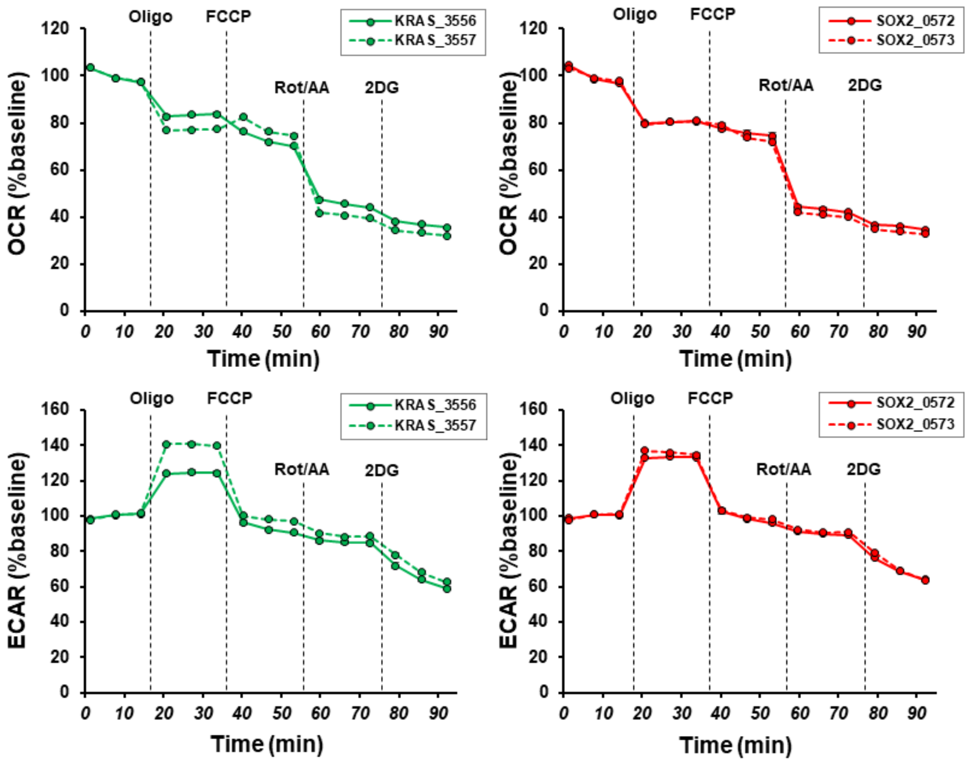

Effects of two different siRNAs on KRAS (KRAS_3556; #SASI_Hs01_00202556, KRAS_3557; # SASI_Hs01_00202557) or SOX2 (SOX2_0572; #SASI_Hs01_00050572, SOX2_0573; # SASI, Hs01_00050573) on mitochondrial and glycolysis function of the 2D cultured SK-mel-24 cells by a Seahorse XFe96 Bioanalyzer were compared. The oxygen consumption rate (OCR) and extracellular acidification rate (ECAR) before drug injection (at the baseline) were determined as 100 %. The fluctuations were then sequentially monitored after the following injections: (i) oligomycin (a complex V inhibitor), (ii) FCCP (a protonphore), (iii) rotenone/antimycin (complex I/III inhibitors), and (iv) 2-DG (a hexokinase inhibitor) (OCR; panel A and ECAR; panel B). Indices of mitochondrial respiration and glycolytic capacity are expressed as % baseline.

Figure S5. qPCR analysis of oncogenic signaling related factors (KRAS, SOX2, MITF, BRAF, FOS and STAT3), ECMs (COL4, COL6, FN, and αSMA), tight-junction related molecule (ZO-1) among 2D and 3D cells obtained from 5 MM cell lines.

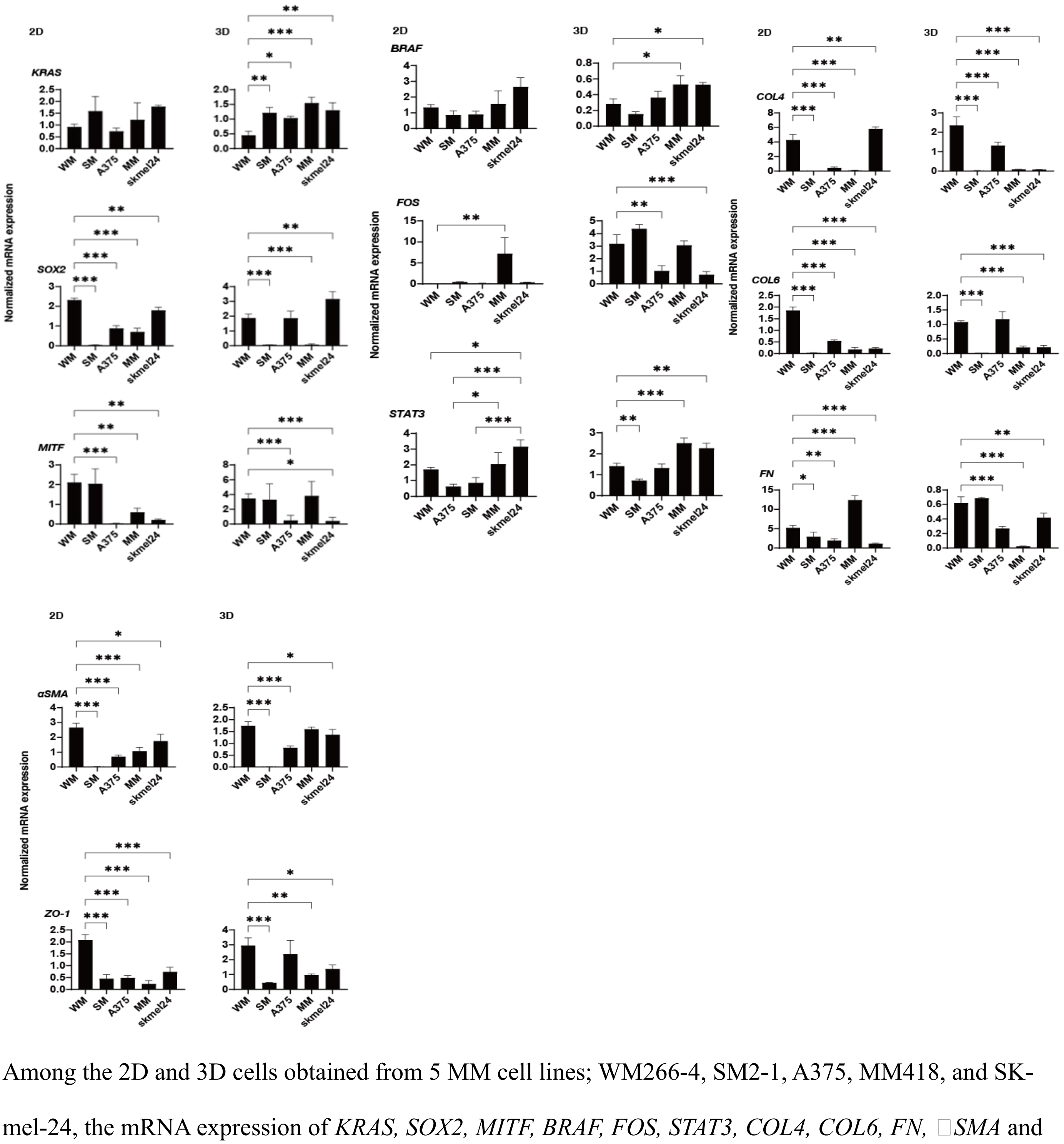

Among the 2D and 3D cells obtained from 5 MM cell lines; WM266-4, SM2-1, A375, MM418, and SK-mel-24, the mRNA expression of *KRAS, SOX2, MITF, BRAF, FOS, STAT3, COL4, COL6, FN, □SMA* and *ZO-1* were evaluated by a qPCR procedure. All experiments were performed in triplicate, each of which used freshly prepared 2D (n=3) and 3D spheroids (n=15-20, total 45-60) in each experimental condition. **P*<0.05, ***P*<0.01, ****P*<0.005.

Figure S6. qPCR analysis of oncogenic signaling related factors (KRAS, SOX2, MITF, BRAF, FOS and STAT3), ECMs (COL4, COL6, FN, and αSMA), tight-junction related molecule (ZO-1) between 2D and 3D cultured A375 and A375DT.

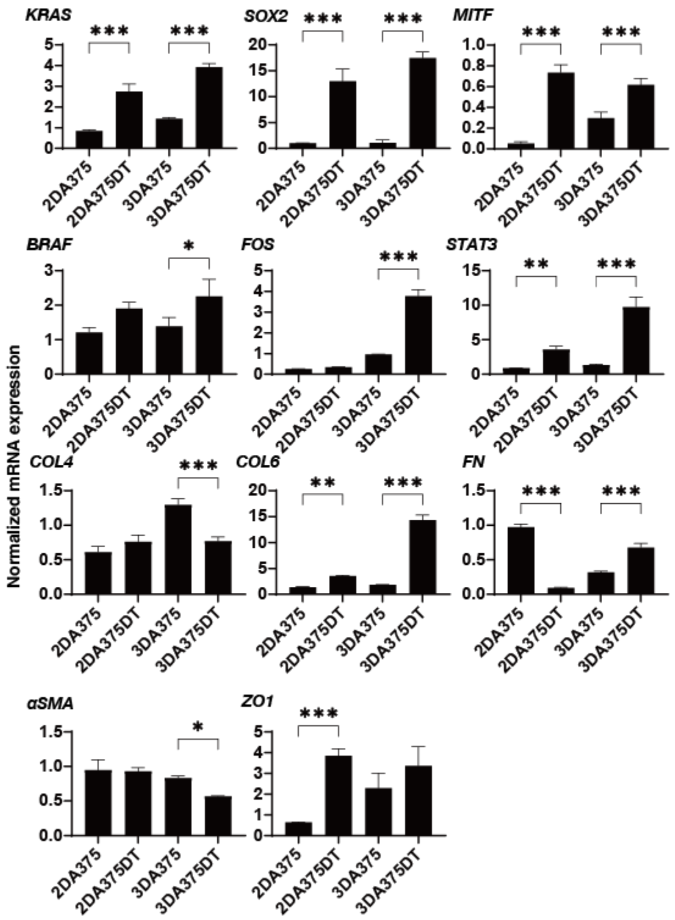

Between the 2D and 3D cultured A375 and A375DT, the mRNA expression of *KRAS, SOX2, MITF, BRAF, FOS, STAT3, COL4, COL6, FN, αSMA* and *ZO-1* were evaluated by a qPCR procedure. All experiments were performed in triplicate, each of which used freshly prepared 2D (n=3) and 3D spheroids (n=15-20, total 45-60) in each experimental condition. *P<0.05, **P<0.01, ***P<0.005.

Supplemental Methods

2D and 3D cell culture of 5 MM cell lines

Five MM cells were each cultured in 2D culture dishes at 37°C in HG-DMEM culture medium supplemented with 8 mg/L d-biotin, 4 mg/L calcium pantothenate, 100 U/mL penicillin, 100 µg/mL streptomycin (b.p. HG-DMEM), 10 % CS and methylcellulose (Methocel A4M) until reaching approximately 90 % confluence. They were then divided into conventional 2D cultures and 3D spheroid cultures. The 2D cultured MM cells were then further maintained with medium changes daily for 7 days. Alternatively, for generating 3D spheroids, after washing with phosphate buffered saline (PBS), the cells were detached by treatment with 0.25 % Trypsin/EDTA, resuspended in the culture medium, and 28 µL of medium containing approximately 20,000 cells were placed into each well of the drop culture plate (# HDP1385, Sigma-Aldrich) (3D/Day 0) as described previously [27, 34]. Thereafter, half of the culture medium was replaced with fresh medium in each well daily until reaching Day 7 [27, 34].

Measurement of real time cellular metabolic functions of various MM cell lines by a Seahorse Bioanalyzer.

Twenty thousand 2D cultured cells, as described above, were placed in wells of a XFe96 Cell Culture Microplate (Agilent Technologies, #103794-100). After centrifuging the plate at 1,000 x g for 10 mins, the culture medium was replaced with 180 µL of Seahorse XF DMEM assay medium (pH 7.4, Agilent Technologies, #103575-100) containing 5.5 mM glucose, 2.0 mM glutamine, and 1.0 mM sodium pyruvate. The assay plates were incubated in a CO_2-free incubator at 37 °C for 1 hour prior to the measurement. OCR and ECAR were simultaneously measured under a 3 min mixing and at 3 mins, the protocols involved measurements at the baseline followed by injections of oligomycin (final concentration: 2.0 µM), carbonyl cyanide p-trifluoromethoxyphenylhydrazone (FCCP, final concentration: 5.0 µM), a mixture of rotenone/antimycin A (final concentration: 1.0 µM), and 2-deoxyglucose (2-DG, final concentration: 10 mM).
